# Enrichment and Molecular Analysis of Breast Cancer Disseminated Tumor Cells from Bone Marrow Using Microfiltration

**DOI:** 10.1371/journal.pone.0170761

**Published:** 2017-01-27

**Authors:** Sreeraj G. Pillai, Peixuan Zhu, Chidananda M. Siddappa, Daniel L. Adams, Shuhong Li, Olga V. Makarova, Pete Amstutz, Ryan Nunley, Cha-Mei Tang, Mark A. Watson, Rebecca L. Aft

**Affiliations:** 1 Washington University School of Medicine, Dept. of Surgery, St. Louis, Missouri, United States of America; 2 Creatv MicroTech, Inc., Rockville, Maryland, United States of America; 3 Creatv MicroTech, Inc., Monmouth Junction, New Jersey, United States of America; 4 Creatv MicroTech, Inc., Chicago, Illinois, United States of America; 5 Creatv MicroTech, Inc., Potomac, Maryland, United States of America; 6 Washington University School of Medicine, Dept. of Orthopedic Surgery, St. Louis, Missouri, United States of America; 7 Washington University School of Medicine, Dept. of Pathology and Immunology, St. Louis, Missouri, United States of America; 8 John Cochran Veterans Administration Hospital, St. Louis, Missouri, United States of America; Universitatsklinikum Hamburg-Eppendorf, GERMANY

## Abstract

**Purpose:**

Molecular characterization of disseminated tumor cells (DTCs) in the bone marrow (BM) of breast cancer (BC) patients has been hindered by their rarity. To enrich for these cells using an antigen-independent methodology, we have evaluated a size-based microfiltration device in combination with several downstream biomarker assays.

**Methods:**

BM aspirates were collected from healthy volunteers or BC patients. Healthy BM was mixed with a specified number of BC cells to calculate recovery and fold enrichment by microfiltration. Specimens were pre-filtered using a 70 μm mesh sieve and the effluent filtered through CellSieve microfilters. Captured cells were analyzed by immunocytochemistry (ICC), FISH for HER-2/*neu* gene amplification status, and RNA *in situ* hybridization (RISH). Cells eluted from the filter were used for RNA isolation and subsequent qRT-PCR analysis for DTC biomarker gene expression.

**Results:**

Filtering an average of 14×10^6^ nucleated BM cells yielded approximately 17–21×10^3^ residual BM cells. In the BC cell spiking experiments, an average of 87% (range 84–92%) of tumor cells were recovered with approximately 170- to 400-fold enrichment. Captured BC cells from patients co-stained for cytokeratin and EpCAM, but not CD45 by ICC. RNA yields from 4 ml of patient BM after filtration averaged 135ng per 10 million BM cells filtered with an average RNA Integrity Number (RIN) of 5.3. DTC-associated gene expression was detected by both qRT-PCR and RISH in filtered spiked or BC patient specimens but, not in control filtered normal BM.

**Conclusions:**

We have tested a microfiltration technique for enrichment of BM DTCs. DTC capture efficiency was shown to range from 84.3% to 92.1% with up to 400-fold enrichment using model BC cell lines. In patients, recovered DTCs can be identified and distinguished from normal BM cells using multiple antibody-, DNA-, and RNA-based biomarker assays.

## Introduction

Disseminated tumor cells (**DTCs**) are detected in the bone marrow (**BM**) of up to 40% of early stage breast cancer patients at the time of diagnosis and are an independent prognostic factor for recurrent disease development[1]. DTCs found in the BM are shed from primary breast cancers and are thought to be intermediaries in the metastatic process[2]. DTCs are rare cells, found with a frequency of about 1 cancer cell per million nucleated BM cells[1], are molecularly heterogeneous, and are often molecularly distinct from their primary tumor of origin[3–5]. Not all patients with BM DTCs, detectable by conventional epithelial markers such as cytokeratin, will develop metastatic disease [6], indicating that DTCs themselves likely differ with regard to further metastatic potential. Molecular analysis of DTCs offers the possibility of identifying specific DTCs with metastatic potential, designing targeted therapies to eliminate these cells, monitoring alterations in tumor cell phenotype and genotype, and predicting therapeutic response[2]. Analysis of DTCs offers information that may not be obtainable in early stage breast cancer patients by studying circulating tumor cells (**CTCs**), which are found with greater rarity in the peripheral circulation[7,8].

Multiple methods have been developed to enrich for rare cells, such as DTCs and CTCs, to allow for their molecular analysis [9]. These methods have been based on the physical and/or molecular properties of the cells. Enrichment techniques include affinity binding approaches by either positive selection (i.e. targeting cell surface antigens such as EpCAM), or negative selection (i.e. by eliminating cells that express the leukocyte specific antigens, such as CD45 (reviewed in [10]). However, conventional antibody-based enrichment methods may not capture a large percentage of DTCs due to their heterogeneity and loss of epithelial antigens, possibly excluding those cells that are important in the metastatic process [11,12]. Other enrichment platforms have been developed for CTCs based on physical properties such as cell size, density, or decreased deformity of the cells (reviewed in [13]). Filtration methods exploit size disparities between cancer cells and normal hematopoietic cells, which allows antigen-independent collection. Microfiltration is rapid and simple and does not require complex instrumentation. It captures both cells and cell clusters as well as allows for the retrieval of viable cells. Several filtration devices are currently available (reviewed in [14]). Most have pore sizes between 7–8 microns which allow flow-through of leukocytes and erythrocytes, that typically measure 6–8 microns in diameter, while cancer cells with diameters of 10 microns or greater are retained[15,16].

Immunocapture for DTCs enrichment has been described [17,18]. However, few of the newer enrichment methods, that are antigen-independent, have been used with BM, which has a more complex cell composition and higher nucleated cell concentration than blood [19]. Robust, reproducible assays for enrichment and characterization of DTCs are required for incorporation of DTC analysis into clinical practice and decision-making. We explored the use of microfiltration, an unbiased method, to improve enrichment and detection of DTCs from the BM of BC patients and to assess its compatibility with several downstream biomarker assay platforms.

## Methods

### Ethics Statement

This study was approved by the Institutional Review Board at Washington University in St. Louis. Informed written consent was obtained from each patient and each healthy volunteer who participated in this study. All patients were enrolled with BM and blood collected between March and June 2014.

### Bone Marrow and Blood Collection

Blood and BM were collected from patients in the operating room during surgical procedures. Blood, 7–10 ml, was initially collected into K2-EDTA or CellSave Cell Preservation tubes (Janssen Diagnostics). BM aspirates were collected into heparinized syringes from normal volunteer posterior iliac crest during orthopedic procedures or from the anterior iliac crest from breast cancer patients with clinical stage II/III disease, prior to treatment initiation, as previously described[4,20]. Collected BM was placed directly into K-EDTA tubes from the syringe without a needle to avoid cell shearing.

### Bone Marrow Processing and Filtering

Nucleated cell counts in BM specimens were determined prior to processing using a Vision CBA cellometer (Nexcelom Biosciences) after dilution with PBS prior to any processing. For spiking experiments, a defined number of SKBR3 breast cancer cells (ATCC) was added at various concentrations to a known number of nucleated BM cells at concentration ratios ranging from 1 to 100 tumor cells per 1x10^6^ nucleated BM cells.

For immunocytochemistry (ICC) and FISH studies, whole BM was diluted 1:10 with PBS, and 7 m of the diluted sample was placed in CellSave Cell Preservation tubes and shipped at ambient temperature to Creatv MicroTech for processing. Specimens were processed within 24 hours of collection. All specimens were pre-filtered using a 70-μm cell strainer (Fisher Scientific) to remove large particles. The effluent was then diluted 1:1 in an equal volume of prefixation buffer (Creatv MicroTech), incubated at room temperature for 15 minutes, and then filtered through CellSieve microfilters (Creatv MicroTech) at a controlled flow rate of 5 ml/minute, generated by negative pressure across the filter[21,22]. As shown in S1 Fig, the CellSeive microfilters consist of a uniform array of 160,000, 7-um pores in a 9 mm diameter area. Captured, fixed cells were then directly used for ICC or eluted and spun onto glass slides for FISH analysis.

For capturing viable cells for RNA-based studies, RBC lysis was performed prior to filtration. Briefly, 7 ml of whole BM was pelleted by centrifugation at 1,200 RPM for 10 minutes, and the supernatant discarded. RBCs were lysed by resuspending the pellet in 40 ml of RBC lysis solution (5 Prime) and incubating for 10 minutes with gentle rocking. The cells were re-pelleted using the same settings and the supernatant was carefully removed. The pellet was then resuspended in a total volume of 14 ml of PBS. As described above, the BM suspension was pre-filtered through a 70-μm mesh sieve to remove bone spicules and the filter was washed once with 1 ml PBS. The total effluent was divided into two equal parts, each filtered through a CellSieve 7-μm pore-size microfilters as previously described [22]. For RNA preparation, filters were place directly into 350 ul of RLT buffer (RNeasy Micro Kit, Qiagen), vortexed vigorously and stored at -80°C until RNA purification. For RISH, cells were eluted from filters as described below.

### Immunocytochemistry and Cell Enumeration

In order to increase accuracy of cell identification, we evaluated the sensitivity and specificity of monoclonal antibodies from various suppliers. A panel of antibodies was previously selected and optimized for identification of circulating cancer cells in peripheral blood while excluding white blood cells ([23,24]. This panel contains a mixture of fluorescent antibodies specific to cytokeratins (CK) 8, 18, and 19 (FITC), EpCAM (PE), and CD45 (Cyanine 5). In this study, the same antibody cocktail was used for both peripheral blood and BM samples. All staining was performed on the filter. Filter membranes with filter-captured cells were post-fixed and permeabilized, according to the manufacturer’s recommendations (Creatv MicroTech), and stained with the fluorescent antibody mixture with the membrane in the filter holder [25]. The filter membrane was subsequently removed from the filter holder and placed directly onto a microscope slide. Ten μL of CellSieve Mounting Solution with DAPI diacetate (Creatv MicroTech) was added to the filter membrane. The filter membrane was covered with a glass coverslip, sealed with nail polish and then examined under fluorescence microscope. A cell was defined as a DTC based on morphology, CK- and/or EpCAM-positive, and CD45-negative[22]. For quantification, all DTCs were counted across the entire filter. To determine the number of residual BM cells, five area images representing 1% of the filter were examined. The average and standard deviation were subsequently calculated, as previously described [25].

### Fluorescence In-Situ Hybridization (FISH)

Fixed cells were recovered from the filters by backwashing. Backwashing was performed by connecting the filter holder to a 10 ml syringe containing 5 ml PBS and flipping the assembly180-degrees, such that the filter side containing cells faced towards a 15 ml collection tube. Five ml of PBS was used to flush the filter. The filter was taken out of the holder assembly and placed in a petri dish and washed two times with 250 ul PBS to collect any additional cells attached to the filter [26]. All released cells were pooled in a 15 ml conical bottom tube and pelleted by centrifugation at 250 RCF for 10 minutes. The cells were resuspended in neutral buffered formalin and incubated at 37°C for 1 hour for fixation. The fixed cells were cytospun on to a positive-charged Superfrost-Plus microscope slide (Fisher Scientific) at 1,000 RPM for 10 minutes. HER-2/neu gene amplification in the transferred cells was examined by FISH with PathVysion HER-2 DNA Probe Kit (Abbott) which contains fluorescence probes specific for the HER-2/neu gene locus (17q11.2-q12) and for the alpha satellite DNA sequence at the centromeric region of chromosome 17 (17p11.1-q11.1). The FISH analysis was performed according to manufacturer’s instructions.

### RNA In-Situ Hybridization (RISH)

Viable cells were recovered from the filters by backwashing as described above, pelleted, then washed in PBMC wash (Advanced Cell Diagnostics), and subsequently suspended in 70% ethanol. Cells collected from one filter were cytospun onto Octospot slides (Thermal Fisher) using a Shannon Cystospin Centrifuge, such that cells from each filter was divided into 8 spots. The slides were then treated sequentially with a peroxidase and protease according to the manufacturer’s recommendations. RNAscope 2.0 HD assays were performed using target probe Hs-HER2 (Advanced Cell Diagnostics). The assays were performed manually according to the manufacturer's instructions. The target probes were hybridized for 2 hours in a hybridization oven at 40°C before performing amplification steps according to the manufacturer. Signals were generated by chromogenic reaction using horseradish peroxidase with 3, 3-diaminobenzidine. The slides were counterstained with hematoxylin and mounted with Cytoseal mounting medium (Richard-Allan Scientific). Positively stained cells were counted manually.

### RNA Preparation

RNA was prepared using RNeasy Micro Kit (Qiagen) according to manufacturer’s protocol. The purified RNA was quantified by NanoDrop ND-2000 spectrophotometer and qualitatively assessed using an Agilent Bioanalyzer.

### RNA Expression Analysis

Quantitative PCR was performed using a microfluidic-based PCR system with 96.96 Dynamic Arrays (Fluidigm Corp.). The cDNAs, prepared from 50-ng of total RNA using Life Technologies High Capacity cDNA Reverse Transcription Kit (Applied Biosystems), were subjected to specific target amplification (**STA**) using TaqMan PreAmp Mastermix (Applied Biosystems). The samples underwent 14 rounds of pre-amplification in the STA process. The cycling program consisted of 10 minutes at 95°C followed by 14 cycles of 95°C for 15 seconds and then 60°C for 1 minute. Each pre-amplified sample was subsequently diluted 1:4 in low EDTA DNA suspension buffer. The sample mixtures were prepared by combining the samples with TaqMan Universal PCR Master Mix (Applied Biosystems) and 20X Gene Expression Sample Loading Reagent (Fluidigm Corp.). The assay mixtures contained 9-μM of each primer and 2-μM of the probe in Dynamic Array Assay Loading Reagent (Fluidigm Corp). The sample and assay mixtures were loaded into appropriate inlets on the primed 96.96 Dynamic Array chip before it was placed on the NanoFlex-4 Integrated Fluidic Circuit Controller for distribution of the sample and assay mixture. The loaded Dynamic Array was then inserted into the BioMark Reverse-Transcription-PCR System. The qPCR program was as follows: 50°C for 2 minutes, 95°C for 2 minutes, 40 cycles of 95°C for 15 seconds and 60°C for 1 minute.

Relative gene expression was calculated using the ddCT method. Each PCR reaction for each sample / gene was repeated in duplicate. Duplicates that varied by more than 1 Ct were discarded. Briefly, Ct values for each gene were normalized to Ct value for *TBP* gene expression in each sample (dCT). A pool of 11 normal bone marrow samples was used to calculate dCT for each gene and the average dCT value for each gene across the normal samples was used as a ‘baseline’. For each sample, relative gene expression was calculated with reference to the average of the normal bone marrow baseline (ddCT), using the expression 2^-ddCT^.

## Results

### Recovery Efficiency of Cancer Cells from BM by Using Microfiltration

**Fig 1:**
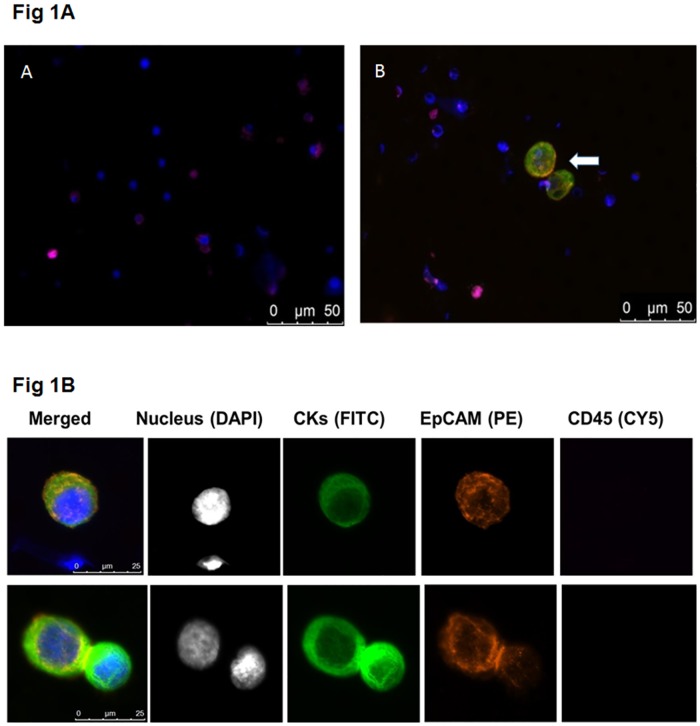
Images of the filter-captured breast cancer tissue culture cells. Fig 1A. (A) Normal bone marrow control cells stained on the microfilter (B) Normal bone marrow cells spiked with SKBR3 breast cancer cells stained on the microfilter. Arrows indicate SKBR3 cells. Fig 1B. Antibody staining of SKBR3 breast cancer cells after filtration. Nuclei are shown as blue in the merged images.

Microfiltration has been optimized for enrichment of CTCs from blood, which has approximately 100-fold fewer mononucleated cells per ml than a typical BM aspirate [27,28]. To evaluate conditions for microfiltration of BM, human breast cancer cell line SKBR3 was diluted into normal BM samples at varying concentrations. For initial experiments, 14 million nucleated BM cells per sample were used for processing by microfiltration. Cancer cell recovery efficiency was estimated by on-filter staining with DAPI and epithelial- or leukocyte- specific antibodies for cytokeratin, EpCAM, and CD45, respectively (Fig 1). Control BM specimens from 5 normal donors demonstrated an absence of CK positive cells or rare CK positive staining cells (0 cells/4 specimens, 4 cells/1 specimen)likely due to non-specific binding of CK to granulocytes or epithelial cell contamination during sample collection or processing.

Recovery of the SKBR3 breast cancer cells averaged 87% (range 85–92%), while **r**esidual mononucleated cells ranged from 26,000 to 41,000 (average 31,900), for an enrichment of approximately 430 fold (range 350–578) with a starting cell number of 14 million cells (Table 1).

**Table 1:** Recovery and fold enrichment of indicated number of SKBR3 tumor cells spiked into an initial sample of 14 million BM cells.

Sample (Bone marrow)*	Input Cancer Cells	Recovered Cancer Cells	Recovery (%)	Residual MNC (No./filter)	Fold Enrichment	Recovery (%)
**1**	0	0	NA	NA	NA	NA
**2**	14	12	85.7	40,000	350	85.7
**3**	140	129	92.1	36,200	386	92.1
**4**	1400	1180	84.3	24,200	578	84.3
Average**				**31,900**	**438**	**87.4**
**SD**				7900		4.19

Abbreviations: MNC-mononucleated cells; NA-not applicable; SD-standard deviation

*Bone marrow from a single normal donor was used from all samples.

** Average was calculated from the 3 spikes samples.

### Immunocytological Analysis of Filtered Cells from Breast Cancer Patients

**Fig 2:**
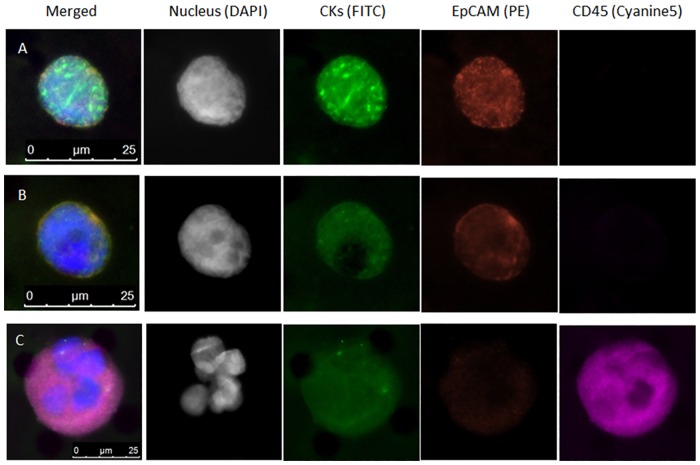
Appearance of filter-captured DTCs from BC patient bone marrow by fluorescent antibody staining. (A, B), Filter-captured DTCs; (C), Hematopoietic precursor cells (HPC). Nuclei are shown as blue in the merged images.

Review of hundreds of cells from patient BM after filtration allowed us to identify six general categories of cells based on nuclear features, nuclear-cytoplasmic ratio, positivity of staining for epithelial markers (EpCAM, CKs) and absence of staining leukocyte marker CD45 (Table 2 and S2 Fig). Categories I and II were CD45-negative cell populations of non-hematopoietic origin, whereas Categories III, IV, V and VI were CD45-positive cell populations of hematopoietic origin. Category I, DTC candidate, were cells scored as true DTCs and were large round cells with increased nuclear-cytoplasmic ratio, negative staining for CD45, and detectable staining for CK8 18, 19 epithelial markers (Fig 2A and 2B). Category II, Questionable DTC, were cells that had morphological characteristics of malignant cells, stained as CD45-negative, but had weak or negative staining for cytokeratins and EpCAM. These cells require additional molecular characterization for confirmation of the cell identity. Category III were cells which were very large and with morphology consistent with typical hematopoietic precursor cells (Fig 2C). The remaining three categories were apoptotic cells with degraded-DNA in nuclei (Category IV), normal blood cells (Category V), and cell fragments or debris (Category VI). Normal BM cells could be readily distinguished from DTCs by their nuclear-cytoplasmic ratios and CD45 staining. Other cancer-associated cells or non-cancer cells could be detected using the antibody cocktail, but could be distinguished from CTCs/DTCs by their morphology[27]. The presence or absence of EpCAM staining alone was not useful as a discriminator. For all analysis, only true DTCs were reported.

**Table 2:** Categories of BM cells after filtration based on morphology and staining.

Categories	Designation	Descriptions	Morphological and Antigenic Characteristics
I	DTC candidates	Disseminated Tumor Cells Candidates	Large round-shaped cells with malignant nuclei, increased nuclear-cytoplasmic ratio; positive staining of CK8, 18, 19 and/or EpCAM, negative staining of CD45
II	Questionable DTC	Questionable Disseminated Tumor Cells Candidates	Different morphologies from white blood cells, CD45-negative, patterns of cytokeratin and EpCAM need to be confirmed
III	HPCs	Hematopoietic precursor cells, consist of many cell types	Very large, round-shaped cells with single- or multi-nuclear, cytoplasm shows weak and smooth staining of all markers including CKs, EpCAM and CD45
IV	dgDNA	Cell degradation, nucleus with degraded-RNA	With or without cytoplasm, smooth staining of DAPI, single or multiple nuclei
V	Normal blood cells	Red blood cells, white blood cells and platelets etc.	White blood cells appear round (lymphocytes) or polymorphonuclear (granulocytes). CD45 positive
VI	Cell Debris	Cell fragments, DNA debris	Fragments of debris from dead cells

BM and blood were analyzed from 8 patients with newly diagnosed clinical stage II/III breast cancer for the presence of DTCs and CTCs prior to any treatment as well as BM from 2 normal healthy volunteers (Table 3). In this set of specimens, the investigators processing the specimens and interpreting the results were blinded to the source of the specimens. Mean follow-up for these patients was 24 months. None of the patients have developed recurrent disease. 2–4 mls of BM was processed for filtering for an average of 51 million cells per filter (range 21–100 million cells) which is the approximate amount of nucleated cells in 7 ml of blood. Neither DTCs nor CTCs were detected in control normal BM or peripheral blood samples. DTCs were detected in all of the eight patient BM samples with numbers ranging from 1–13. CTCs were detected in 2 of 6 patient samples with 1 cell detected per sample. Increased detection of cancer cells in BM compared to blood is consistent with other reports [7].

**Table 3:** Patient characteristics and enumeration of DTCs and CTCs.

Pt ID	Age	Hist.*	ER	PR	Her2	Grade	Tumor size (cm)	LN	Mets	BM cells filtered (No. X10^6^)	DTC (No.)	CTC (No.)
6837	32	IDC	Neg	Pos	Neg	3	3.9	Neg	no	62	4	0
9037	35	IDC	Pos	Pos	Neg	2	4.7	Pos	no	22	5	0
5324	67	IDC	Pos	Neg	Neg	3	4.5	Pos	no	55	1	na
2219	62	IDC	Neg	Neg	Neg	2	2.9	Neg	no	21	13	1
3641	44	IDC	Neg	Neg	Neg	3	8.5	Pos	no	35	10	0
6079	46	IDC	Neg	Neg	Neg	3	4.5	Pos	no	27	9	0
4717	54	ILC	Pos	Neg	Neg	1	4.0	Pos	no	67	4	1
7914	67	IDC	Neg	Neg	Neg	3	1.5	Pos	no	100	5	Na
Control	72	Nl	na			na				84	0	0
Control	74	Nl	na			na				100	0	Na

*Abbreviations are: Hist.-histology; ER-estrogen receptor; PR-progesterone receptor; LN-clinical lymph node status; Mets-metastatic disease development; No.-number; Nl-normal; na-not applicable

### Fluorescence In Situ Hybridization (FISH) and RNA-ISH

**Fig 3:**
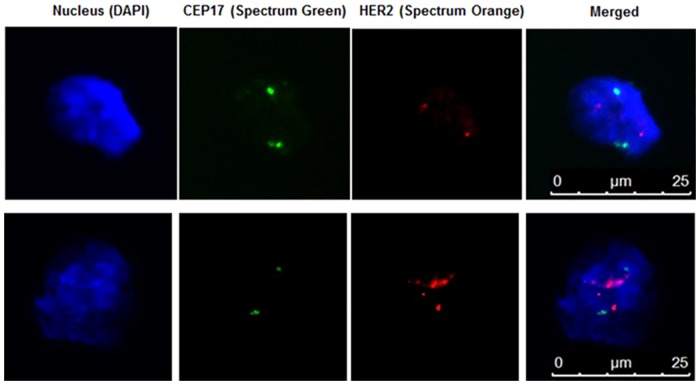
Her2-FISH analysis of DTCs from breast cancer patient BM. Top Row: DTC with normal copy number of CEP17 and Her2. Bottom Row: DTC with normal copy number of CEP17 and increased copy number of Her2.

We also performed FISH for HER2 gene amplification, a predictive marker, on BM cells from breast cancer patients after filtration. Fig 3 shows an example of a BM DTC from a BC patient with amplification of the HER2 gene. Significantly, the primary tumor from this same patient (5324) was scored as negative for HER2 gene amplification. Discordance in HER2 gene amplification between primary tumors and DTCs has been previously documented and has possible treatment implications for targeted therapeutics [29–31]

**Fig 4:**
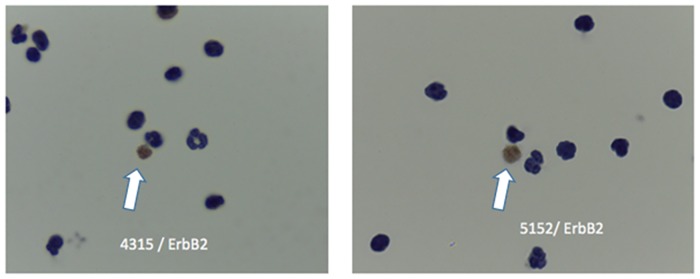
RNA-In situ hybridization for ERBB2 gene expression. BM from 2 breast cancer patients after filtration analyzed by RNA-ISH for Her-2 expression. Her2/ERBB2 RNA-ISH positive cells are brown (arrows).

Although cells that have been fixed prior to filtration can be easily visualized on the filters by immunohistochemistry or DNA-based FISH, fixative treatment results in degradation of RNA. Thus we also optimized elution of viable cells from the filters to evaluate gene-expression based biomarkers. Using a backwashing methodology, we were able to elute an average of 1.7x10^5^ cells (range 1.4-75x10^4^ cells) per filter with an average starting number of 70 million cells (range 4-16x10^7^ cells, N = 10 specimens, S1 Table). Staining of the filters after backwashing revealed no residual cells. RNA-ISH for Her2 was performed on BM from breast cancer patients and normal BM. An example of DTC detected by RNA-ISH for Her2/ERBB2 is shown in Fig 4. There was no detectable staining for probes in normal BM (data not shown)

### RNA Purification and mRNA Expression Analysis

**Fig 5:**
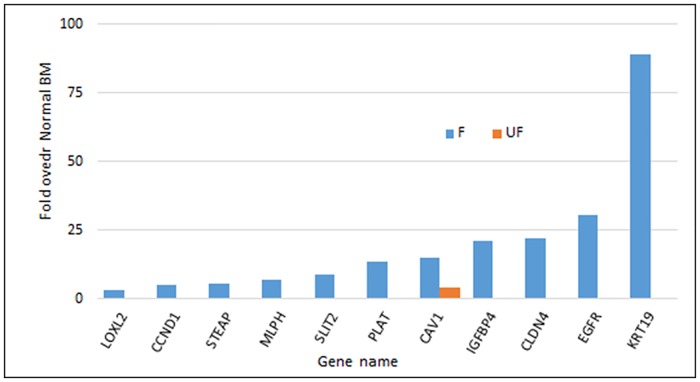
Expression of DTC associated transcripts in filtered and unfiltered BM specimens. Expression of 11 genes in normal BM samples spiked with 20 MDA-MB-231 cells per million nucleated BM cells compared to unspiked normal BM (NBM) as shown in x-axis. RNA expression was measured in either filtered (F) or unfiltered (UF) BM samples. Gene expression was determined by qRT-PCR on a Fluidigm platform. The actual fold values over normal BM are depicted.

We found that RNA quantity and quality could be improved and was more consistent between preparations by performing RBC lysis before BM filtration. Using a high-throughput, qRT-PCR platform, we analyzed the expression of 11 genes (CAV1, CCDN1, CLDN4, EGFR, IGFBP4, KRT19, LOXL2, MLPH, PLAT, SLIT2 and STEAP) in filtered and unfiltered BM, that we and others have found to be expressed by DTCs and breast cancer cells lines, but absent in normal BM [29,32]. Normal BM was spiked with SKBR3 breast cancer cells at varying concentrations and a total of 37 million normal BM cells filtered. All 11 genes were detected in filtered BM samples spiked at the lowest dilution of 1:1,000 with SKBR3 cells, but only 7 of the genes (CLDN4, EGFR, LOXL2, MLPH, PLAT, SLIT2 and STEAP) could be detected in the corresponding, unfiltered samples (S3 Fig). At the highest dilution of cancer cells of 1:50,000 spiked SKBR3 cells, which is more biologically relevant, expression of all 11 DTC biomarker genes were still detected in filtered BM samples, but only 1 gene (CAV1) was detectable in the unfiltered samples (Fig 5). To evaluate the quality and yield of RNA isolated from filtered cells using patient specimens, we performed RNA isolation and quality assessment from a consecutive set of 66 early stage breast cancer patient BM samples. 2–4 ml of BM was used and the average number of BM cells filtered was 63 million (range 1.6-20x10^7^). RNA yield was an average of 135 ng per 10 million BM cells filtered (range 224–3270 ng) with an average RIN of 5.33 (range 1–9.1).

## Discussion

In this study, we have demonstrated the effective use of a sized based filtration-method for DTC enrichment from BC patient BM aspirate specimens. While eliminating biases associated with antigen-capture based approaches, we demonstrate that this method allows for efficient recovery and enrichment of DTCs, which may then be readily utilized for sensitive downstream detection methodologies such as immunocytochemical, *in situ* hybridization, or qPCR-based gene expression assays. Though many technologies have been used to enrich for CTCs, few of these have been tested using BM for DTCs (summarized in[33]) and we believe that this is the first report of using size filtration for DTC enrichment.

In early stage breast cancer patients, the presence and persistence of DTCs in the BM have been associated with a poor prognosis [34,35]. DTCs have been used as a surrogate for clinical outcome after chemotherapy[36]. Enumerated DTCs and CTCs are both prognostic for breast cancer recurrence, but whether both are equivalent in predicting disease recurrence in early stage breast cancer patients is not clear and may be dependent upon the enumeration and enrichment techniques used for each [7,37–39].

In our study employing filtration enrichment, we detected DTCs in all patient BM specimens analyzed. None of these early stage breast cancer patients developed a relapse within the follow-up of 24 months. We are confident that the identified cells were DTCs based on both morphological and immunofluorescence antibody staining characteristics. The method used for enrichment of DTCs may result in varying recovery rates and sensitivity. For example, ficoll density gradient centrifugation is commonly used for enrichment of DTCs followed by ICC staining and has been recommended by the German Consensus group of Senology for enrichment of mononucleated cells from BM aspirates [40]. However, ficoll enrichment can be associated with loss of mononucleated cells [41–43] with the cell recovery rates between 15–30% [41,42]. Immunomagnetic separation for enrichment of DTCs has not been recommended because of its high cost and lack of superiority over Ficoll enrichment. In our study, we were able to recover >85% of cancer cells spiked into normal BM using microfiltration technology for enrichment. While we are able to demonstate the feasibility of using microfilitration to capture DTCs, due to the small patient sample size and short follow-up, we were unable to demonstrate a clear correlation between DTC detection and patient outcome. Further studies with larger patient samples would or detection of specific genes associated with DTCs would be needed.

Potentially, expression of epithelial cells associated genes, non-specific binding of antibodies or certain pathological conditions can lead to increased enumeration rate/higher background. In this study, the antibody cocktail used for detection had been optimized for detection of epithelial cells while minimizing background. Using this antibody cocktail, we observed 4 cells which met criteria for DTC in only one normal BM, which also was the first normal BM processed using microfiltration. However, to fully study illegitimate expression, specimens would need to be analyzed from a broader patient population with differing clinical backgrounds.

DTCs have been found to differ from the primary tumor both phenotypically and genetically [3]. For example about 10–20% of patients with Her2-negative primary tumors will have Her2-positive DTCs [29–31]. This can be due to both the evolution of cells during their sojourn into the BM or the presence of a pre-existing, occult sub clonal population of cells in the primary tumor that is simply not detected by conventional diagnostics.

Ideally, molecular characterization of DTCs would allow for the identification of those cells associated with disease recurrence as well as provide a rational basis for selection of targeted therapies. Whether therapeutically targeting DTCs based on their molecular profile will improve disease outcome remains to be determined, though there are ongoing clinical trials based on targeting Her2 expressing DTCs in patients with Her2-negative tumors which are addressing this question (For example **NCT01779050**). Incorporation of targeted DTCs therapies based on their molecular profiles into clinical practice would require a robust, reproducible assay for analyzing these cells in an unbiased manner.

In this study, we have optimized enrichment of DTCs from the BM of breast cancer patients using size-based microfiltration. Most of the enrichment technologies for rare cells have been developed for CTCs from blood which is a less complex medium than BM. Using size-filtration, we found that there was approximately 90% recovery of input tumor cells with approximately 400 fold enrichment, and that cells could be recovered for sensitive downstream analyses (Summarized in Table 4). The retention of 30–40,000 WBC in our experiments is consistent with the level of retained mononucleated cells from microfiltered blood [44], as is the recovery of 85% of the input cancer cells filtered from blood [44]. Ficoll-hypaque enrichment of DTCs and CTCs has been commonly used, and results in an approximately 3.8 fold depletion of mono-nucleated cells [45] and detection of approximately 1 DTC per million mononucleotide BM cells based on immunocytochemical detection of cytokeratin expression. Using filtration, we achieved an approximately 400 fold depletion of mononucleated cells and were able to detect DTCs with a >50-fold increased sensitivity using ICC compared to standard ICC detection of DTCs.

**Table 4:** Recovery efficiency of breast cancer cells from bone marrow spiking experiments.

	Calculation	Results
**Capture Efficiency**	CC Captured/ CC actual	87%
**Enrichment**	(CC captured/WBC captured) /(CC actual/WBC initial)	350–400 fold
**Purity**	CC captured/(CC+WBC) captured	0.3%
**Throughput**	Cells/unit time	15 x10^6 cells/ minute

Abbreviations: CC-cancer cell

We found that high quality RNA could be isolated from cells after filtration, which allowed us to test RNA based gene expression assays. Using PCR, expression of DTC associated genes could be detected at a concentration of 20 cells per million BM cells, which was the lowest concentration that we tested. Moreover, we have been able to visualize expression of specific genes from individual filtered cells, using RNA-based in situ hybridization.

Examination of hundreds of cells from BC patient BM allowed us to classify cells into five major categories based on their staining and morphological features. Codifying the classification of cells found in the BM will allow investigators to compare results as well as to identify those cells which are important in the development of metastatic disease.

In a small sample set of eight clinical stage II/III breast cancer patients, using identical enrichment and enumeration for both DTCs and CTC analysis, we detected DTCs in all BM by ICC after filtration, while CTCs were detected in the blood of only 2 of these patients. Other investigators have compared the prognostic significance of CTCs and DTCs and have reported that not all patients with CTCs had DTCs and vice versa [46–48]. However, none of these studies have enumerated CTCs and DTCs using identical technologies for detection. Standardization for enumeration and molecular characterization of DTCs as well as CTCs will be required for determining the prognostic significance. This will allow the identification of those subpopulations of DTCs that will generate overt metastases and lead to the design of specific targeted therapy to the cells.

Though microfiltration is an antigen-independent enrichment of DTCs that allows the recovery of viable cells, there are several limitations. First, pure populations of DTCs are not recovered. Based on spiking experiments, we estimate the purity to be about 0.3% (Table 4). Thus detection methods need to rely on DTC specific features. Second due to the manipulation of the cells, morphology may not always maintained. Despite this, microfiltration is very reproducible and allows the rapid enrichment of DTCs without the requirement for sophisticated equipment.

In summary, we have optimized a size-based filtration method for enriching for cancer cells from bone marrow. This technique results in high recovery and the capture of viable cells that can be used for down-stream analysis requiring intact RNA and DNA for molecular profiling. Given the ease and reproducibility of size filtration, we envision that this can be readily incorporated into clinical practice for the enrichment and molecular analysis of DTCs.

## Supporting Information

S1 Fig Scanning electron microscope image of CellSieve microfilters with 7-μm diameter pores in a uniform array. (PPTX)

S2 Fig Representative microfiltered BM cells. (PPTX)

S3 Fig Detection of DTCs-associated gene expression by PCR in BM. (PPTX)

S1 Table Retrieval of BM Cells after elution from filters. (DOCX)
