# Supplementary material for: Enrichment and Molecular Analysis of Breast Cancer Disseminated Tumor Cells from Bone Marrow Using Microfiltration
Source: PLoS One. 2017 Jan 27;12(1):e0170761. doi: 10.1371/journal.pone.0170761 (PMC5271341; doi:10.1371/journal.pone.0170761)
Supplement: S1 Fig — (PPTX) [file pone.0170761.s001.pptx]

## Slide 1
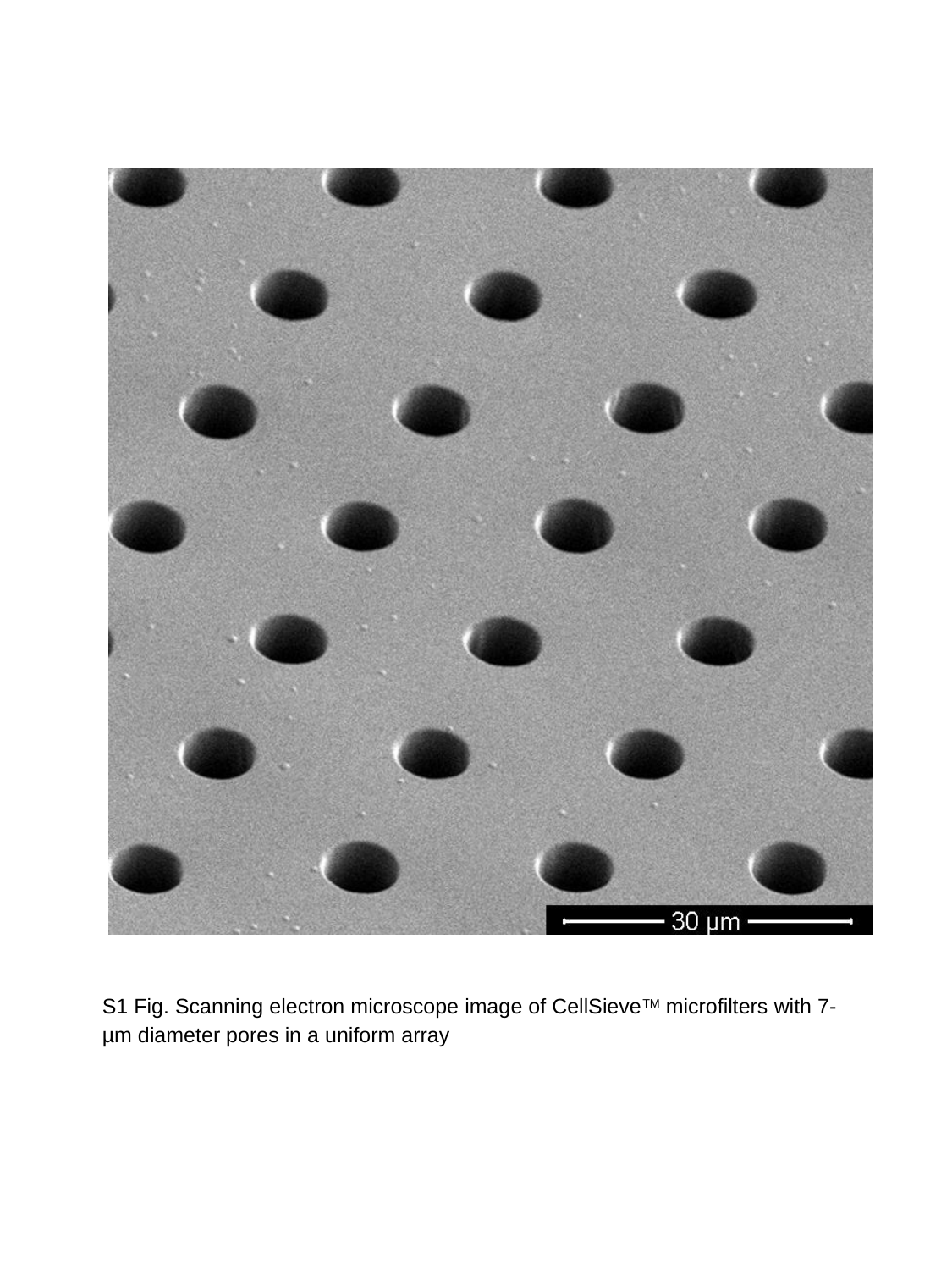

S1 Fig. Scanning electron microscope image of CellSieveTM microfilters with 7-µm diameter pores in a uniform array
