# Supplementary figures and images for: Enrichment and Molecular Analysis of Breast Cancer Disseminated Tumor Cells from Bone Marrow Using Microfiltration
Source: PLoS One. 2017 Jan 27;12(1):e0170761. doi: 10.1371/journal.pone.0170761 (PMC5271341; doi:10.1371/journal.pone.0170761)

## Slide 1
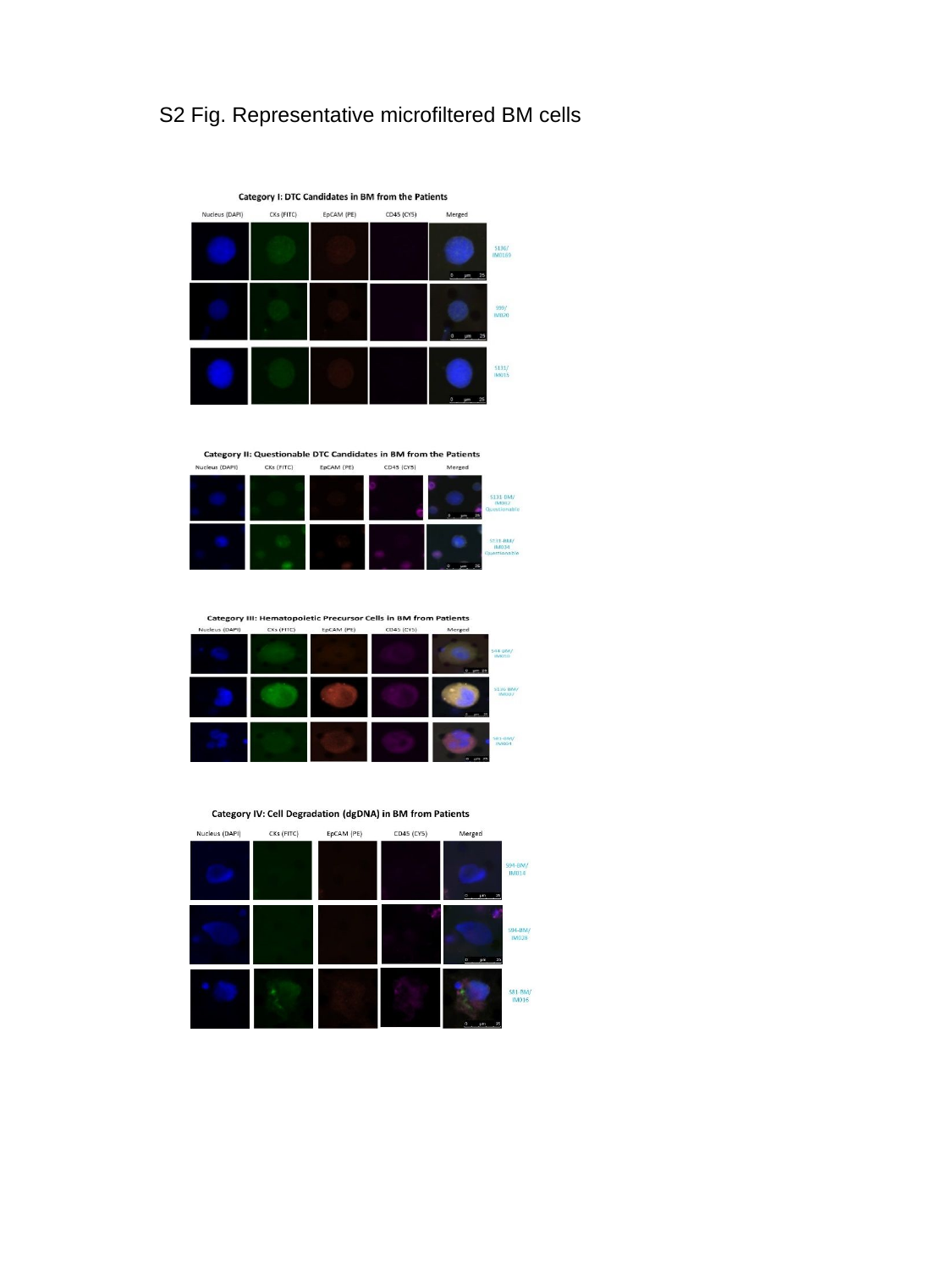

S2 Fig. Representative microfiltered BM cells

## Slide 2
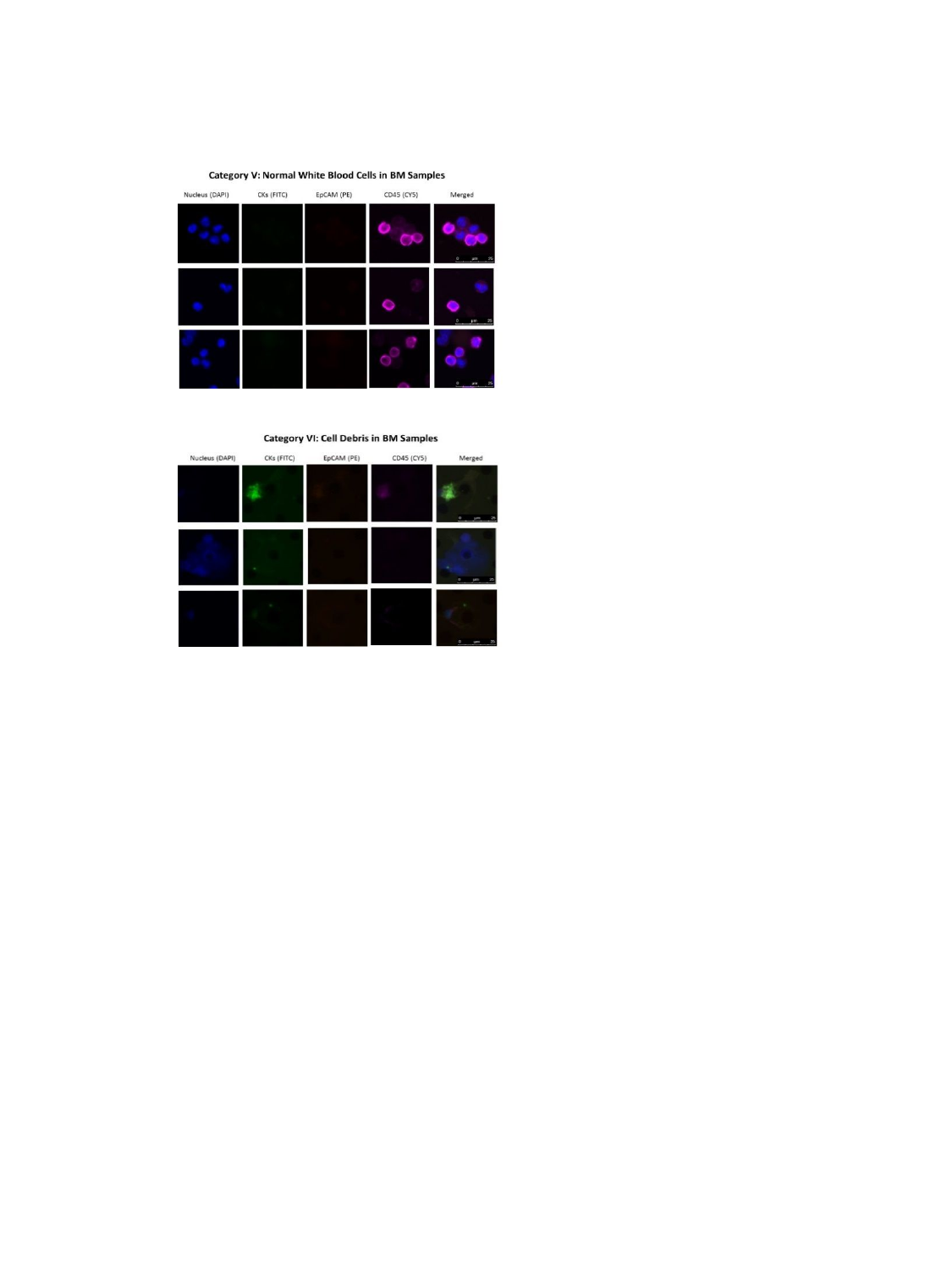

Supplement: S2 Fig — (PPTX) [file pone.0170761.s002.pptx]

## Slide 1
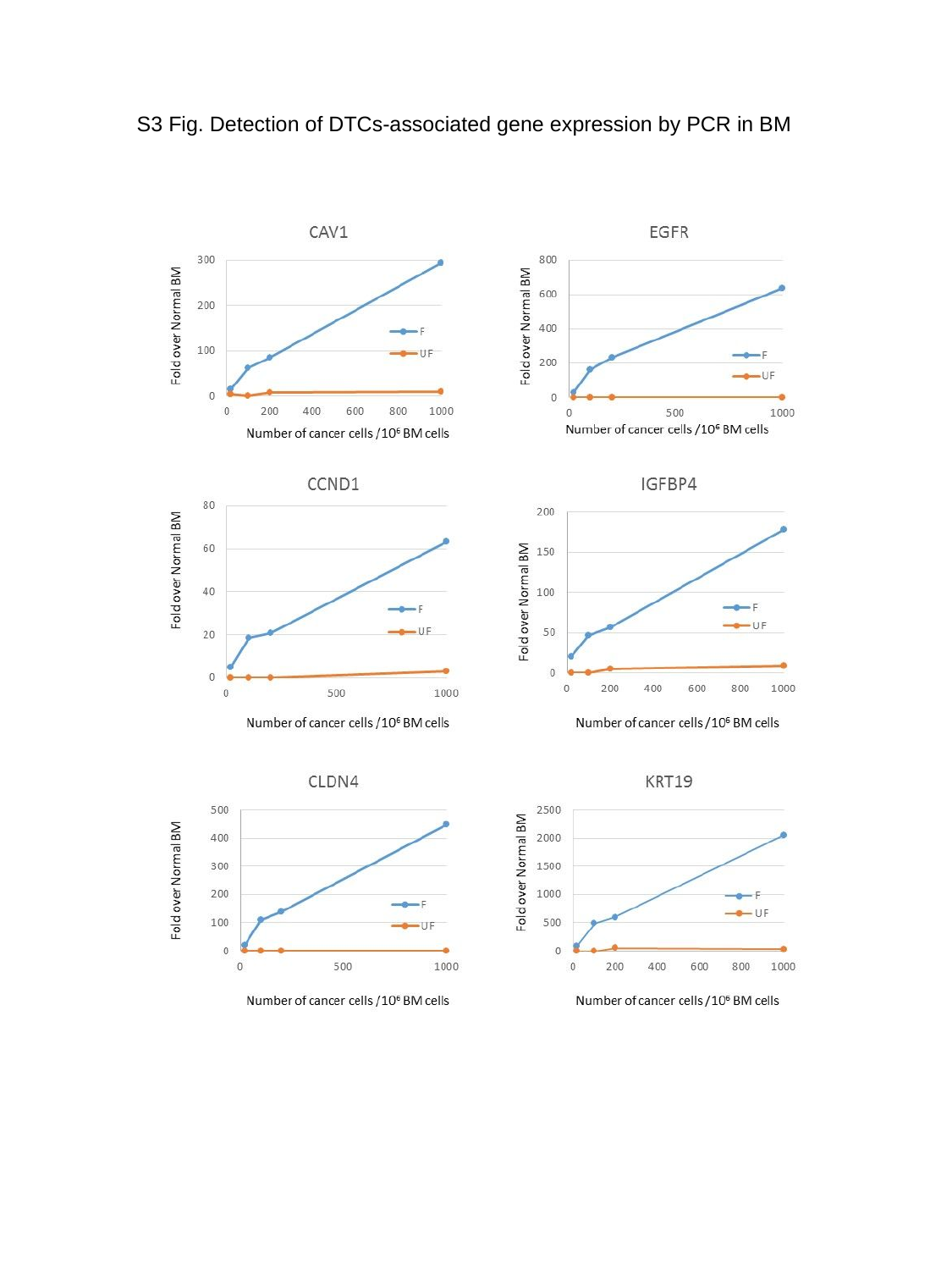

S3 Fig. Detection of DTCs-associated gene expression by PCR in BM

## Slide 2
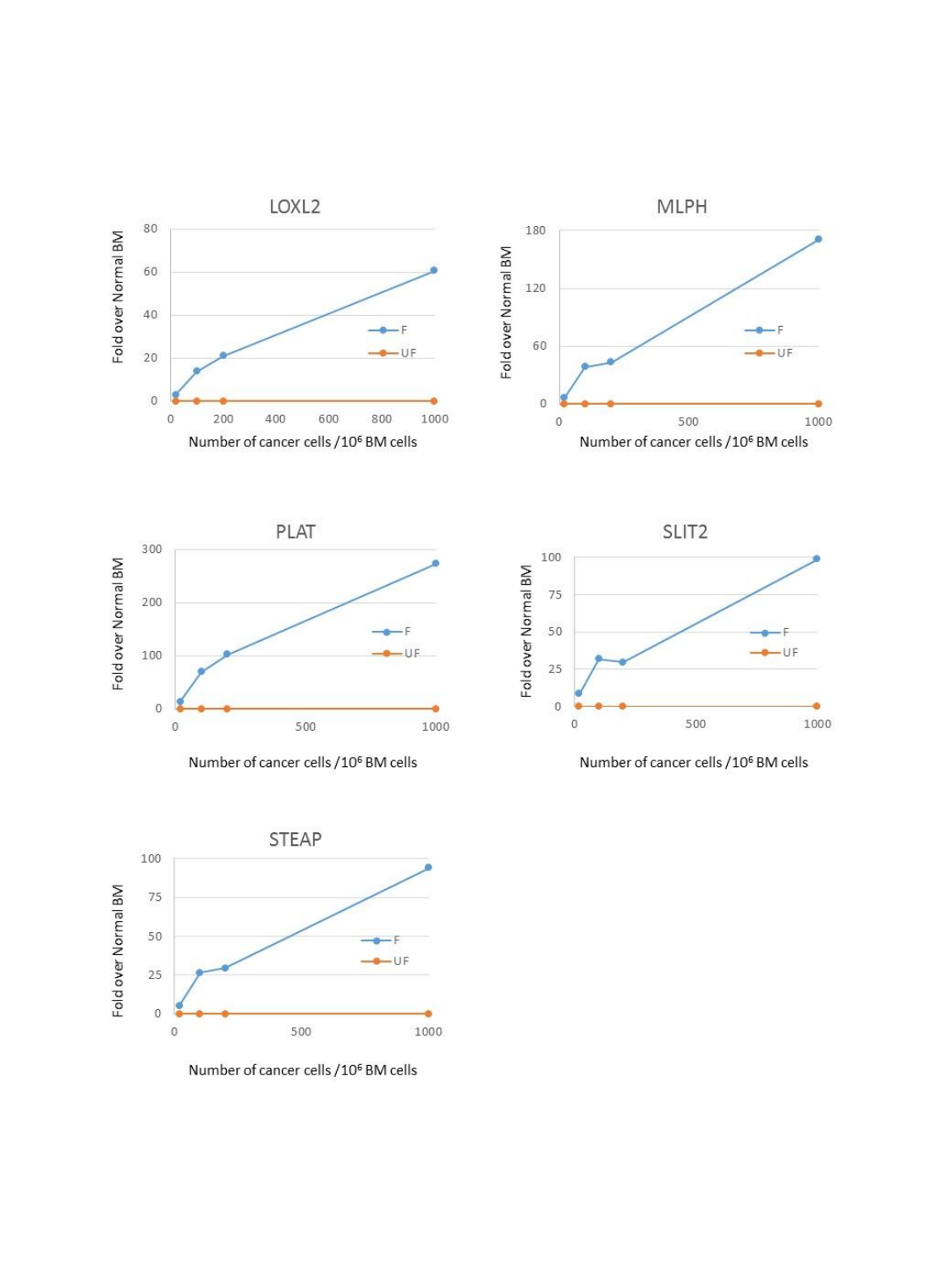

Supplement: S3 Fig — (PPTX) [file pone.0170761.s003.pptx]
