# Supplementary material for: Enrichment and Molecular Analysis of Breast Cancer Disseminated Tumor Cells from Bone Marrow Using Microfiltration
Source: PLoS One. 2017 Jan 27;12(1):e0170761. doi: 10.1371/journal.pone.0170761 (PMC5271341; doi:10.1371/journal.pone.0170761)
Supplement: S1 Table — (DOCX) [file pone.0170761.s004.docx]

S1 Table. Retrieval of BM Cells after elution from filters

| **Sample ID** | **Number of BM cells filtered x10^7** | **Number of BM cells retrieved from filter** | **Volume of BM filtered (ml)** |
| --- | --- | --- | --- |
| 1129 US | 16.2 | 128000 | 4 |
| 1129 S | 16.2 | 740000 | 4 |
| 1130 | 3.75 | 132000 | 4 |
| 1131 | 1.00 | 14200 | 4 |
| 1132 S BW | 8.05 | 25600 | 4 |
| 1132 BW | 8.05 | 101600 | 4 |
| 1138 S BW | 4.18 | 96382 | 2.5 |
| 1138 US BW | 4.18 | 11663 | 2.5 |
| 1139 S FBW | 4.35 | 351440 | 3 |
| 1139 US FBW | 4.35 | 79200 | 3 |
| Average | 7.031 | 168,009 |  |
